# How much can reticulate evolution entangle plant systematics? Revisiting subfamilial classification of the Malvatheca clade (Malvaceae) on the basis of phylogenomics

**DOI:** 10.3389/fpls.2025.1717745

**Published:** 2026-01-23

**Authors:** Gustavo Luna, Lucas Costa, Flávia Fonseca Pezzini, Nisa Karimi, Joeri Sergej Strijk, Jefferson Carvalho-Sobrinho, Matheus Colli-Silva, André Marques, Gustavo Souza

**Affiliations:** 1 Laboratory of Plant Cytogenetics and Evolution, Department of Botany, Federal University of Pernambuco, Recife, Pernambuco, Brazil; 2 Laboratory of Evolutionary Ecology and Plant Genomics, Institute of Biology, State University of Campinas, Campinas, Brazil; 3 Royal Botanic Garden Edinburgh, Edinburgh, United Kingdom; 4 Department of Botany, University of Wisconsin – Madison, Madison, WI, United States; 5 Alliance for Conservation Tree Genomics, Kuala Lumpur, Malaysia; 6 Universidade Federal do Vale do São Francisco, Petrolina, Brazil; 7 Universidade Federal Rural de Pernambuco, Recife, Brazil; 8 Laboratory of Plants Morphotaxonomy and Evolution, Department of Botany, Federal University of Pernambuco, Recife, Pernambuco, Brazil; 9 Royal Botanic Gardens, Richmond, United Kingdom; 10 Department of Chromosome Biology, Max Planck Institute for Plant Breeding Research, Cologne, Germany

**Keywords:** bombacoideae, malvoideae, matisioideae, phylogenomics, polyploidy, reticulate evolution

## Abstract

Reticulate evolution (RE), involving hybridization and related processes, generates network-like rather than strictly bifurcating relationships among lineages and can obscure phylogenetic relationships. Detecting ancient hybridization is particularly challenging, as genomic signals may erode over time. The Malvatheca clade (Malvaceae), marked by multiple paleopolyploidy events since it’s estimated origin 66 my, offers a useful model for examining RE. Its three subfamilies—Bombacoideae (with high chromosome numbers, mostly trees), Malvoideae (lower chromosome numbers, mostly herbs), and the recently described Matisioideae—show unresolved relationships, with several taxa of uncertain placement. We conducted a phylogenomic analysis of 69 Malvatheca species via complete plastomes, 35S rDNA cistrons, nuclear low copy genes and comparative repeatome data. Most of the datasets consistently resolved four clades: (I) Bombacoideae, (II) Malvoideae, (III) Matisioideae, and (IV) a heterogeneous assemblage including representatives of Malvoideae, Matisioideae and several *incertae sedis* taxa. Chromosome numbers were negatively correlated with repeatome diversity: Bombacoideae presented higher counts but lower repeat diversity, possibly reflecting slower repeat evolution associated with woody growth forms. In contrast, clades III and IV showed marked heterogeneity in both chromosome number and repeat composition, which is consistent with a reticulate origin. Overall, our results show evidence of ancient hybridization and polyploidy in shaping Malvatheca evolution. These results highlight that reticulation and genome dynamics, rather than taxonomic boundaries alone, are central to understanding the diversification of Malvatheca.

## Introduction

Reticulate evolution (RE) refers to processes in which genetic material is exchanged between lineages, producing network-like rather than strictly bifurcating phylogenetic relationships (Arnold, 1997; Gontier, 2015). It can arise through hybridization, horizontal gene transfer, or endosymbiosis, and challenges the traditional tree-like view of evolution (Mallet, 2007). Reticulation also complicates classification by blurring taxonomic boundaries and generating conflicting phylogenetic signals. In plants, where hybridization is common, genetic and phenotypic distinctions may be obscured, leading to uncertain or misleading taxonomic boundaries (Mallet, 2007).

Polyploidy (whole genome duplication, WGD), the presence of multiple complete genomes in a species, is closely linked to reticulation and is a recurrent feature of angiosperm evolution (Bowers and Paterson, 2021). Allopolyploidization, i.e., hybridization followed by genome doubling, has been recognized as a major driver of plant diversification (Paterson et al., 2004; Jiao et al., 2011; Vekemans et al., 2012). Notably, a burst of polyploidization events in flowering plants occurred near the Cretaceous–Paleogene (K–Pg) boundary (~66 Mya), coinciding with a mass extinction event (Vanneste et al., 2014; Fawcett and Van de Peer, 2010). The clustering of independent WGDs across angiosperms during this period suggests that polyploidy promoted survival and diversification by providing raw material for genetic novelty (Fawcett et al., 2009; Wood et al., 2009).

Studying RE in ancient lineages remains difficult because signals of hybridization and polyploidy are often eroded over time (Zhang et al., 2025). Additionally, disentangling processes such as incomplete lineage sorting, horizontal transfer, or genetic erosion is challenging (Mallet, 2007), and modeling ancient reticulation requires large datasets and sophisticated analyses. High-throughput sequencing has made these investigations feasible (Dodsworth et al., 2019). Target enrichment methods such as Hyb-Seq enable the analysis of thousands of low-copy nuclear loci (Karbstein et al., 2022), whereas genome skimming can recover plastid genomes (tracing maternal inheritance), ribosomal DNA (biparental nuclear signal), and repetitive elements such as satellites and transposable elements (Dodsworth, 2015; Cavallini et al., 2019). Repeat abundance and sequence similarity offer complementary, alignment-free tools for phylogenetic reconstruction (Dodsworth et al., 2019; Vitales et al., 2020).

The Malvatheca clade (Malvaceae), which originated near the K–Pg boundary (~66 Mya; Hernández-Gutiárrez and Magallon, 2019; Cvetković et al., 2021), serves as a system for investigating these processes. It comprises Bombacoideae (17 genera, ~160 species), Malvoideae (78 genera, ~1,670 species), and the recently recognized Matisioideae (three genera, ~138 species) (Baum et al., 2004; Carvalho-Sobrinho et al., 2016; Cvetković et al., 2021; Colli-Silva et al., 2025). Matisioideae, formerly the tribe Matisieae, was elevated to subfamily rank by Colli-Silva et al. (2025) after consistent recovery of its monophyly, despite its morphological distinctiveness. While Matisioideae is a well-supported lineage, its position—sister to Bombacoideae or to a recircumscribed Malvoideae—remains poorly resolved, even with phylogenomic data. Malvoideae includes widely cultivated herbs such as cotton, hibiscus, and okra (Baum et al., 2004), whereas Bombacoideae consists mainly of tropical trees, including ecologically and culturally significant species such as the baobab (*Adansonia* L. sp.) and the kapok tree (*Ceiba pentandra* (L.) Gaertn.) (Wickens, 2008; Zidar and Elisens, 2009). Several genera, such as *Ochroma* Sw. and *Chiranthodendron* Larreat., placement remain uncertain due to conflicting morphological and molecular evidence. This phylogenetic uncertainty, coupled with the clade’s history of polyploidy, makes Malvatheca a valuable system for studying reticulate evolution in ancient lineages.

The subfamilies within Malvatheca appear to have followed contrasting genomic trajectories shaped by postpolyploid diploidization. Bombacoideae is predominantly composed of species with high chromosome numbers (2n = 86–276; Costa et al., 2017), whereas Malvoideae ranges from 2n = 10–130, with a modal value near 2n = 16 (Tate et al., 2005). Genomic studies have reported signals of reticulate allopolyploidization in Malvatheca and his consequences to their phylogentic relationships (Hernández-Gutiérrez et al., 2022; Sun et al., 2024; Yang et al., 2025; Zhang et al., 2025), but its consequences for genome architecture, or diversification still poorly known. To address this question, we analyze ribosomal DNA, plastome and repeatome data of 22 Bombacoideae, 34 Malvoideae, 7 Matisioideae, and 6 *incertae sedis* taxa under a comparative phylogenomic framework. Specifically, we ask the following questions: (i) Is there evidence of reticulate evolution in the group, and is it associated with taxa of uncertain placement? (ii) Are there repeatome signatures linked to ancient WGDs? (iii) Does lifeform correlate with the distinct diploidization pathways observed in Bombacoideae and Malvoideae?

## Materials and methods

### Data acquisition and repeatome characterization

We analyzed 69 species of the Malvatheca clade, including 34 Malvoideae, 22 Bombacoideae, 7 Matisioideae, and 6 *incertae sedis*. Two *Theobroma* species (Bytnerioideae) were included as outgroups. Species names and NCBI accession codes (when available) are listed in **Table 1**. Target-capture sequencing was used to expand the representation of Bombacoideae, following Costa et al. (2021). Not all species had reads with sufficient reads quality/amount to proceed with all analysis. Thus it was not possible to perform repeatome characterization based on most of the Matisioideae species (except *Pragmotheca mammosa* and *Quararibea funerabis*).

**Table 1:** Species and accessions used.

Bombacoideae	*n*	1C (PG)	Access	Approach
*Adansonia digitata* L.	84	1.7	SRX19282309	WGS
*Adansonia grandidieri* Baill.	44	0.76	SRX24110595	WGS
*Adansonia gregorii* F.Muell.	44	0.68	SRX24110598	WGS
*Adansonia madagascariensis* Baill.	44	0.69	SRX24110597	WGS
*Adansonia perrieri* Capuron	42	0.71	SRX7064913	HybSeq
*Adansonia rubrostipa* Jum. & H.Perrier	44	0.65	SRX24110596	WGS
*Adansonia suarezensis* H.Perrier	44	0.74	SRX7064911	HybSeq
*Adansonia za* Baill.	44	0.63	SRX7064903	HybSeq
*Bombax ceiba* L. 1	46	1.62	SRX20143449	WGS
*Bombax ceiba* L. 2	46	1.62	SRX7064904	HybSeq
*Ceiba aesculifolia* (Kunth) Britten & Baker f.	–	–	PRJNA579976	HybSeq
*Ceiba pentandra* (L.) Gaertn. 1	43	1.75	SRX20143754	WGS
*Ceiba pentandra* (L.) Gaertn. 2	43	1.75	Pezzini et al., 2021	HybSeq
*Ceiba* sp*eciosa* (A.St.-Hil., A.Juss. & Cambess.) Ravenna	43	1.25	SRX18461125	WGS
*Eriotheca macrophylla* (K.Schum.) A.Robyns	138	4.77	SRX14481707	HybSeq
*Gyranthera amphibiolepis* W.Palacios	–	–	SRX14481737	HybSeq
*Huberodendron swietenioides* (Gleason) Ducke	–	–	SRX14481725	HybSeq
*Neobuchia paulinae* Urb.	–	–	SRX14481682	HybSeq
*Pachira aquatica* Aubl	46	2.3	PRJNA1288544	HybSeq
*Pentaplaris davidsmithii* Dorr & C.Bayer	–	–	SRX14481702	HybSeq
*Pseudobombax croizatii* A.Robyns	–	–	SRX7064905	HybSeq
*Rhodognaphalon schumannianum* A.Robyns *(Rhodognaphalon mossambicense* (A.Robyns) A.Robyns)	–	–	PRJNA579976	HybSeq
*Scleronema micranthum* (Ducke) Ducke	–	–	SRX7064906	HybSeq
*Spirotheca rosea* (Seem.) P.E.Gibbs & W.S.Alverson	44	–	SRX14481726	HybSeq
Malvoideae				
*Abelmoschus esculentus* (L.) Moench	65	1.23	SRX21378022	WGS
*Abutilon amplum* Benth.	–	–	SRX5462903	WGS
*Abutilon fruticosum* Guill. & Perr.	21	–	ERX12138327	HybSeq
*Abutilon theophrasti* Medik.	21	1.4	SRX9130922	WGS
*Alcea rosea* L.	21	–	ERX9653860	WGS
*Althaea officinalis* L.	21	–	SRX2855182	WGS
*Decaschistia byrnesii* Fryxell	–	–	ERX7170161	HybSeq
*Gossypium arboreum* L.	13	1.7	SRX5759203	WGS
*Gossypium herbaceum* L.	13	1.7	SRX9217821	WGS
*Gossypium raimondii* Ulbr.	13	0.9	SRX341462	WGS
*Hampea nutricia* Fryxell	13	–	SRX14481653	HybSeq
*Hibiscus brachysiphonius* F.Muell.	–	–	SRX5462993	WGS
*Hibiscus × rosa-sinensis* L.	42	–	ERX9653895	WGS
*Hibiscus syriacus* L.	40	–	SRX529352	WGS
*Hibiscus trionum* L.	28	–	SRR17686673	WGS
*Howittia trilocularis* F.Muell.	–	–	ERX7170167	Hybseq
*Kokia cookei* O.Deg.	12	0.57	SRX24024016	WGS
*Kokia drynarioides* (Seem.) Lewton	12	0.6	SRX3304973	WGS
*Kokia kauaiensis* (Rock) O.Deg. & Duvel	12	0.57	SRX24024021	WGS
*Kosteletzkya pentacarpos* (L.) Ledeb.	17	–	SRX12399957	WGS
*Lawrencia densiflora* (Baker f.) Melville	–	–	SRX5463054	WGS
*Lavatera assurgentiflora* subsp. *glabra* (*Malva assurgentiflora* subsp. *glabra* (Kellogg) M.F.Ray)	–	–	SRX23987976	HybSeq
*Malva sylvestris* L.	21	1.5	ERX3149988	WGS
*Malva moschata* L.	21	1.4	ERX3149982	WGS
*Malva pusilla* Sm.	21	–	ERX5310142	WGS
*Napaea dioica* L.	14	8.5	SRX30129087	WGS
*Pavonia schiedeana* Steud.	–	–	SRX14481669	HybSeq
*Pavonia hastata* Cav.	28	–	ERX7170168	HybSeq
*Pavonia triloba* Guill. & Perr.	–	–	ERR5165833	HybSeq
*Pavonia urens* Cav.	–	–	ERR7622263	HybSeq
*Sida cardiophylla* F.Muell.	–	–	SRX5462742	WGS
*Sida clementii* Domin	–	–	SRX5462743	WGS
*Sida* sp*inosa* L.	7	1	SRX5462745	WGS
*Thespesia populneoides* (Roxb.) Kostel.	13	–	SRX706235	WGS
Matisioideae				
*Matisia alchornifolia* Triana & Planch.	–	–	ERR7622266	HybSeq
*Matisia ochrocalyx* K.Schum.	–	–	ERR7622272	HybSeq
*Phragmotheca ecuadorensis* W.S.Alverson	–	–	ERR7622267	HybSeq
*Phragmotheca mammosa* W.S.Alverson	–	–	SRX14481713	HybSeq
*Quararibea asterolepis* Pittier	–	–	SRR15016217	HybSeq
*Quararibea duckei* Huber	–	–	ERR14030174	HybSeq
*Quararibea funebris* (La Llave) Vischer	–	–	SRX14481652	HybSeq
Incertae sedis				
*Lagunaria patersonia* (Andrews) G.Don	–	–	SRX14481717	HybSeq
*Camptostemon schultzii* Mast.	–	–	SRX14481714	HybSeq
*Alyogyne huegelii* (Endl.) Fryxell	32	–	SRX14481721	HybSeq
*Chiranthodendron pentadactylon* Larreat.	–	–	SRX14481650	HybSeq
*Fremontodendron mexicanum* Davidson	–	–	SRX25649159	HybSeq
*Ochroma pyramidale* (Cav. ex Lam.) Urb.	42	2.2	SRX14481665	HybSeq
Outgroup (Byttnerioideae)				
*Theobroma cacao* L.	20	0.4	SRX23903616	WGS
*Theobroma grandiflorum* (Willd. Ex Spreng.) K.Schum.	20	0.51	SRX17564460	WGS

Repeatome analyses were performed with the RepeatExplorer2 pipeline implemented in the Galaxy server (https://repeatexplorer-elixir.cerit-sc.cz; Novák et al., 2020), which clusters reads on the basis of sequence similarity. Two approaches were applied: (i) individual analyses, where each species was analyzed separately to characterize its repetitive fraction, and (ii) comparative analysis, where concatenated reads from all species were analyzed jointly to compare repeat composition across lineages.

For individual analyses, ~0.1× genome coverage per species was used (**Table 1**). For species with unknown genome sizes, values were estimated on the basis of closely related taxa. For the comparative analysis, reads were normalized to ~0.05× per species, yielding a total of 61,288,798 reads. In both cases, clustering was performed using 90% sequence similarity and 55% minimum overlap. The proportions of repeat lineages were calculated as the number of reads per cluster relative to the total number of reads analyzed, excluding chloroplast and mitochondrial sequences identified as potential contaminants.

### Correlations between karyotypic, genomic and ecological traits

Following Schley et al. (2022), we treated the genome as a “community” and repetitive element lineages as “species,” allowing the application of community ecology metrics to genome composition. Repeat diversity was quantified via the Shannon diversity index (H; Shannon, 1948), which measures the probability that two randomly chosen repeat copies belong to the same lineage. Higher H values indicate greater repeat diversity.

For each species, diversity indices were calculated from repeat abundances obtained from the RepeatExplorer individual analyses. To test whether repeat diversity was associated with chromosome number, we performed a Pearson correlation between log_10_;-transformed Shannon index values and log chromosome counts across Malvatheca species via the stats package in R (R Core Team, 2019).

To further examine potential ecological correlates, we compiled chromosome numbers and growth habits (herbaceous *vs*. woody) for 84 species from the literature. Relationships between habit and chromosome number were visualized with boxplots constructed in PAST 4 (Hammer et al., 2001).

### Plastome, rDNA, low copy nuclear genes and repeat-based phylogenies

Plastomes and ribosomal DNA (rDNA) sequences were assembled for all sampled species using a reference-based mapping approach in Geneious v6.0.3 (Kearse et al., 2012). *Theobroma cacao* (NC_014676.2) served as the reference for plastome assembly, while sequence JQ228369.1 was used for rDNA. Reads were mapped to their respective references using the “Map to reference” function, and consensus plastome sequences were aligned with MAFFT (Katoh and Standley, 2013).

The raw reads from each of the 69 species were mapped against 44,343 gene sequences from the *Ceiba pentandra* genome assembly (Shao et al., 2024) using the “Map to reference” function. These mapped genes were filtered based on mapping quality, phylogenetic resolution, and a coverage threshold requiring the gene to be present in at least 67 species. This filtering process yielded four genes suitable four phylogenetic analysis (CpUnG0025.1, CpUnG0488.1, CpUnG0595.1, CpUnG1740.1). A maximum likelihood (ML) phylogenetic tree was inferred from these four genes, the plastome and the rDNA alignments using IQ-TREE software (v3.0.1). The resulting tree was visualized in FigTree (https://tree.bio.ed.ac.uk/software/figtree/). Topological incongruences between the plastid, rDNA, and nuclear gene trees were visualized using a circular tanglegram generated with the circlize package (Gu et al., 2014) in R. Phylogenetic inference based on repeat abundances was performed following Dodsworth et al. (2015). The abundances of repeat classes obtained from comparative repeatome analyses were treated as quantitative characteristics. Parsimony analyses were conducted in PAST 4 (Hammer et al., 2001).

### Reticulate evolution

Reticulate evolution was assessed by constructing phylogenetic networks in PhyloNet 3.8.2 (Wen et al., 2018). The input for this analysis consisted of the individual tree topologies from the complete plastome, rDNA, and four nuclear loci. We employed a two-step approach: (1) estimation of an underlying ML species tree from the set of input gene trees using the DeepCoalCount_tree algorithm (H_0_); and (2) inference of a phylogenetic network by iteratively adding reticulations to this species tree with the DeepCoalCount_network algorithm to estimate the number of hybridization events (H_1_, H_2_, H_3_ etc.). The inferred network was visualized using SplitsTree v6.4.17 (Huson and Bryant, 2006). To independently validate the crosslinking events proposed by network inference, we performed Patterson’s D-statistic test (ABBA-BABA test) based on nucleotide site patterns (Green et al., 2010; Durand et al., 2011). For this, we used information from all loci (plastomes + rDNA + low-copy nuclear genes) analyzed here for representatives of the Malvatheca clade. We calculated the D value for specific taxonomic quartets ((P1, P2), P3, O) designed to test each gene flow hypothesis, where a significant deviation from zero (D > 0) indicates an excess of shared derived alleles between P2 and P3, consistent with introgression.

## Results

### Comparison between plastidial, rDNA and low-copy nuclear genes topologies

**Figure 1:**
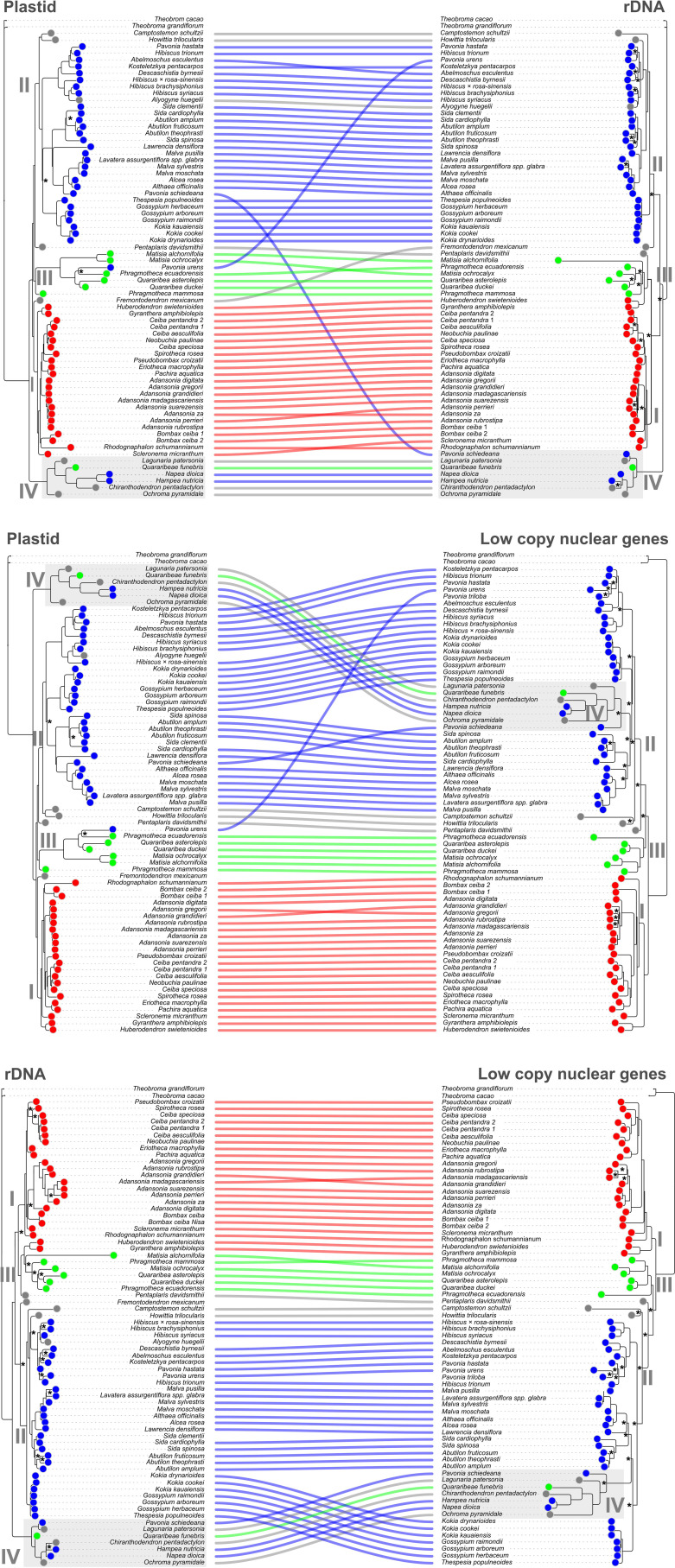
Phylogenetic relationships within the Malvatheca clade (Malvaceae) inferred from plastome, nuclear rDNA and low copy nuclear genes. Tip labels are color-coded by taxonomic group: Malvoideae (blue), Bombacoideae (red), Matisioideae (green), and *incertae sedis* (gray). Lines connect the same taxa across the topologies, highlighting congruences and conflicts. Black asterisks stand for support values lower than 70.

Clade I (Bombacoideae) was well-supported across all three topologies, with one key exception: *Huberodendron swietenioides* + *Gyranthera amphibiolepsis* were absent from the rDNA topology, appearing instead within Clade III (**Figure 1**). Furthermore, on the plastid topology, the *incertae sedis* species *Fremontodendron mexicanum* was resolved as the earliest-diverging lineage of this clade (**Figure 1**).

Clade II, also well-supported, primarily comprises Malvoideae species. Exceptions include *Pavonia urens*, which was placed in Clade III (Matisioideae) on the plastid topology, and *Pavonia schiedeana*, which appeared in Clade IV in the low-copy nuclear and rDNA topologies (**Figure 1**). This clade also includes the *incertae sedis* species *Camptostemon schultzii*, *Howittia trilocularis*, and *Pentaplaris davidsmithii*; the latter was placed here in the plastid topology, while *Fremontodendron mexicanum* was placed here in the rDNA topology (**Figure 1**).

Clade III (Matisioideae) was consistently well-supported (**Figure 1**). However, several species from other groups appeared within it depending on the topology: the *incertae sedis* species *Pentaplaris davidsmithii* and the Bombacoideae species *H. swietenioides* and *G. amphibiolepsis* on the rDNA topology; the Malvoideae species *Pavonia urens* on the plastid topology (**Figure 1**). On the other hand, the Matisioideae specie *Quararibea funebris* was placed in another clade (IV) across all analyses (**Figure 1**). Remarkable, the backbone of the subfamilies was variable depending on the dataset, with clade III (Matisioideae) being sister to Bombacoideae in rDNA topology or sister to Malvoideae in plastidial and low-copy nuclear gene topologies (**Figure 1**).

Clade IV is morphologically unclear composed of three *incertae sedis* species (*Chiranthodendron pentadactylon*, *Lagunaria patersoniana* and *Ochroma pyramidale*), one Matisioideae (*Q. funebris*), and two Malvoideae species (*Hampea nutricia* and *Napea dioica*). Additionally *Pavonia schiedeana* was an exception, appearing in this clade only in the low-copy nuclear and rDNA topologies, not in the plastid topology (**Figure 1**). In the low copy nuclear genes topology, these species were placed within the Clade II. The incongruence and relationship details are present in **Supplementary Figures 1**, **2**.

### Reticulate evolution in Malvatheca

**Figure 2:**
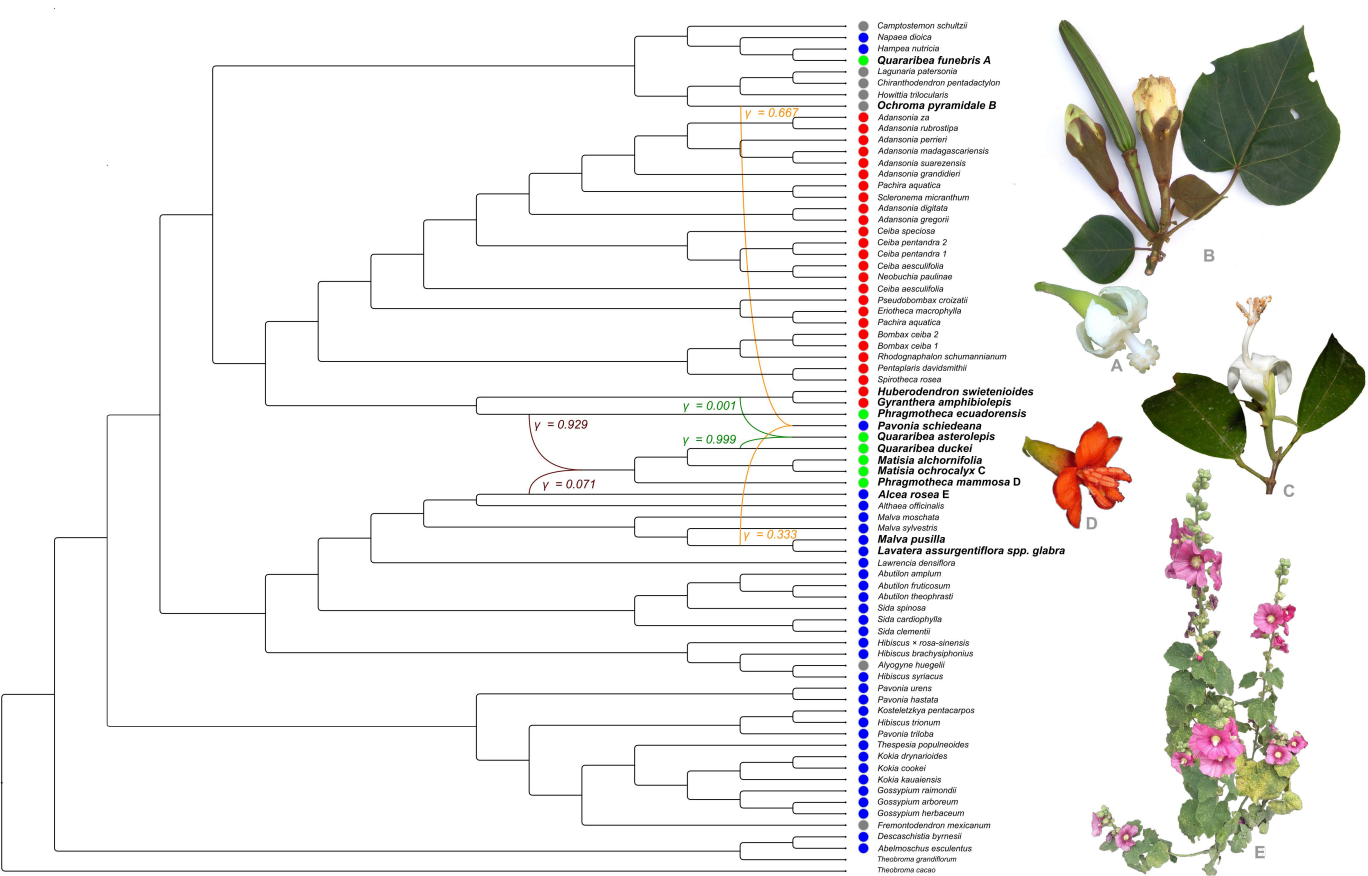
The optimum phylogenetic network inferred from single gene trees of the four nuclear low copy genes + rDNA + plastomes performed for Malvatheca clade (Malvaceae) using PhyloNet analyses.

By assuming positional changes in nuclear and plastidial topologies as evidence of reticulate evolution, our data revealed a high complexity among plastidial, rDNA, and low-copy nuclear gene topologies (**Figure 1**). Major topological incongruences involved the placement of two *Pavonia* species. *P. urens* was resolved within Clade III in the plastid topology, while *P. schiedeana* was placed in Clade IV in both the low-copy nuclear and rDNA topologies. The PhyloNet analysis provided strong evidence for reticulate evolution in Malvatheca. The DeepCoalCount function perform a species tree with a ‘total number of extra lineages score’ of 695.0. Subsequent testing the distinct reticulation hypotheses (H_1_–H_4_) using the DeepCoalCount_network algorithm yielded the following scores: H_1_ = 517.0, H_2_ = 497.0, H_3_ = 456.0, and H_4_ = 466.0 (The lower this score, the less conflict there is between the tested loci). Thus, H3 was selected and this network reveal three statistically supported reticulation events within the Malvatheca clade (**Figure 2**): (i) A hybrid origin of *Pavonia schiedeana* Steud. involving the cross between the lineages of *Malva pusilla* Sm. + *Lavatera assurgentiflora* subsp. *glabra* (Cockerell) Guilliams clade (inheritance proportion, γ = 0.333) and *Ochroma pyramidale* (Cav. ex Lam.) Urb. (γ = 0.667); (ii) a hybrid origin of *Quararibea asterolepis* Pittier by the cross between the lineages *Huberodendron swietenioides* (Gleason) Ducke + *Gyranthera amphibiolepis* W.Palacios clade (γ = 0.001) and *Quararibea duckei* Huber (γ = 0.999); (iii) a ancient hybridization between ancestors lineages of *Phragmotheca ecuadorensis* W.S.Alverson (γ = 0.929) and *Alcea rosea* L. (γ = 0.071), which gave rise to the entire clade III (**Figure 2**). Network inference (PhyloNet) under Maximum Parsimony and Maximum Likelihood criteria rejected strict bifurcation in favor of a network with three hybridization events. We independently validated the two main events using D-statistics (ABBA-BABA test) based on nucleotide site patterns. We detected strong evidence of introgression between the *Pavonia* lineage and the Malveae clade (D = 0.61), as well as between the tribe Matisieae (*Quararibea*) and the genus *Huberodendron* (D = 0.54), confirming that the observed topological discordance is not merely the result of incomplete lineage sorting (ILS), but rather of significant ancient gene flow. On the other hand, the reticulation at the base of the Matisioideae clade showed D = 0.05, with the distribution of shared (ABBA) and unshared (BABA) alleles being almost perfectly symmetrical (19 to 17), suggesting a scenario of Incomplete Lineage Sorting (ILS).

### Repeatome diversity in the Malvatheca clade

**Figure 3:**
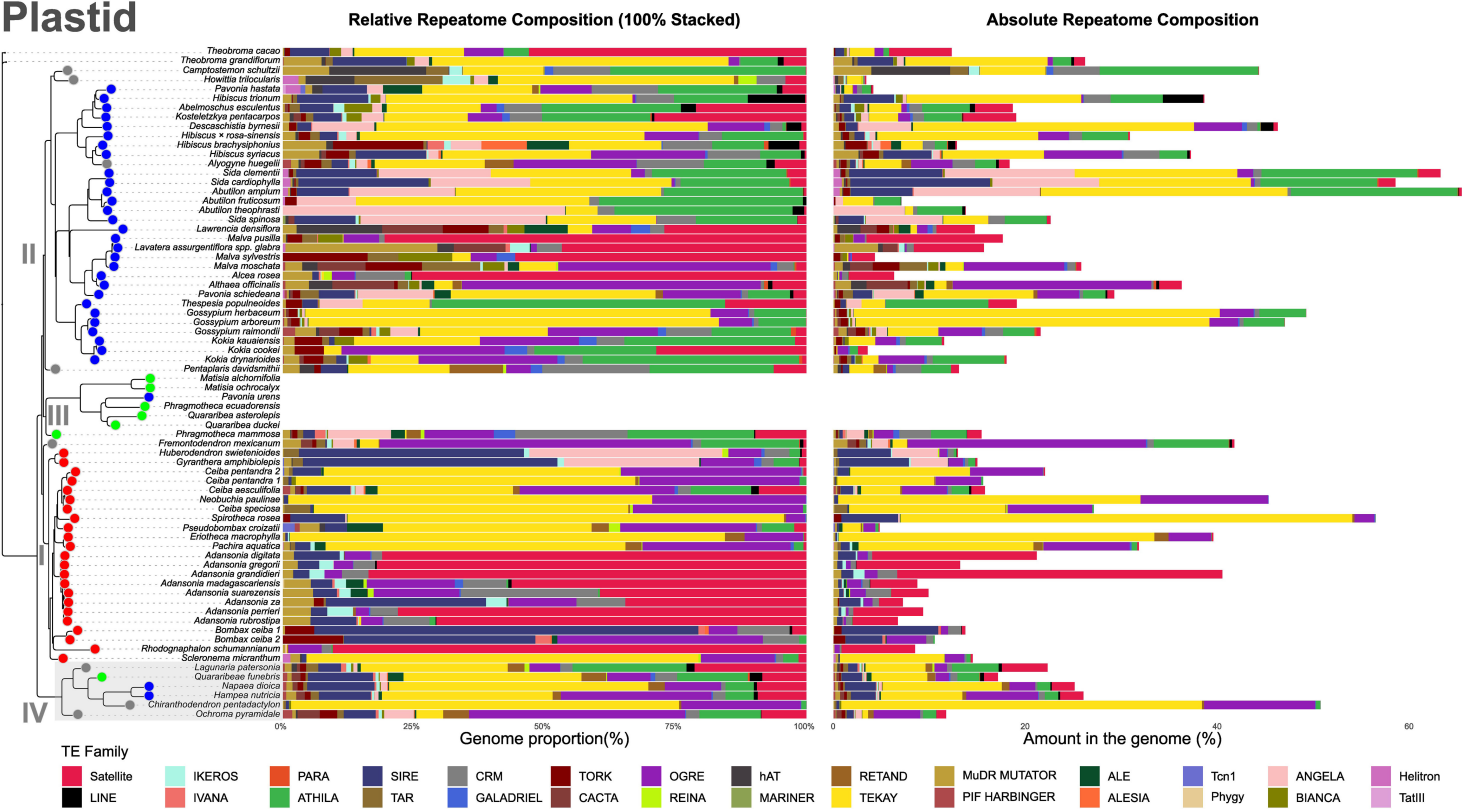
Repeatome composition of the Malvatheca clade. The plastome-based phylogeny (left) is shown alongside the repeat profiles for each species (right). Stacked bars indicate the relative proportions of major repeat classes (normalized to 100%), whereas dots represent their estimated absolute genomic abundance (Mb).

In the individual repeatome analyses, the sequencing depth ranged from 40,572 reads in *Bombax ceiba* (accession 2) to 2,633,822 in *Hibiscus syriacus* (**Supplementary Table 1**). The proportion of repetitive DNA varied widely, from 4.8% in *Pseudobombax croizatii* A. Robyns to 56.4% in *Spirotheca rosea* (Seem.) P.E.Gibbs & W.S. Alverson (Bombacoideae), and from 3.4% in *Howittia trilocularis* to 65.4% in *Abutilon amplum* Benth. (Malvoideae) (**Figure 3**; **Supplementary Table 2**).

Both subfamilies were dominated by Class I transposable elements, particularly LTR retrotransposons of the *Ty3-type* superfamily. Their abundance ranged from 0.5% in *Malva sylvestris* L. to 50% in *Chiranthodendron pentadactylon* Larreat. (**Supplementary Table 2**). Within Bombacoideae, Tekay elements predominated across four genera, whereas Ogre elements also occurred at moderate to high levels. In Malvoideae, Tekay and Ogre were generally present in moderate proportions, with a few species showing elevated abundances of Athila elements. Among *Ty1-copia* elements, Angela and SIRE were the most widespread across both subfamilies, with SIRE being particularly enriched in *Sida cardiophylla* Domin. and one *Bombax ceiba* accession (**Figure 3**; **Supplementary Table 3**). The satellite DNA content also varied considerably, from 0.02% in *Gossypium arboreum* L. to 33.9% in *Adansonia grandidieri* Baill. Bombacoideae, especially *Adansonia*, tended to have relatively high satellite DNA abundances (4.9–33.8%). Despite this variation, no genomic synapomorphies based on repeat presence/absence were detected that clearly distinguished Bombacoideae from Malvoideae (**Figure 3**; **Supplementary Tables 2**, **3**).

Comparative repeatome analysis revealed limited sharing of repetitive DNA clusters between subfamilies. Within Bombacoideae, intrageneric similarity was high: *Adansonia* species shared the most repeat clusters, and *Pseudobombax croizatii* and *Pachira aquatica* Aubl. Both genera presented elevated proportions of *Ty3-type* Ogre and CRMs. In contrast, Malvoideae species presented highly divergent repeatomes, with even congeneric taxa (e.g., *Malva sylvestris* and *M. moschata* L.) sharing few clusters (**Supplementary Figure 3**; **Supplementary Table 4**).

The repeat-based phylogeny recovered three major clades but with generally weak support (**Supplementary Figure 4**; **Supplementary Table 4**). Clade III showed extensive mixing, grouping representatives of all subfamilies without resolution. Several genera were not recovered as monophyletic, including *Adansonia* (Bombacoideae) and *Abutilon*, *Sida*, *Hibiscus*, and *Malva* (Malvoideae). Three Bombacoideae species (*Phragmotheca mammosa*, *Gyranthera amphibiolepis* W. Palacios, *Huberodendron swietenioides* (Gleason) Ducke) and four Malvoideae species (*Camptostemon schultzii* Mast., *Pentaplaris davidsmithii* Dorr & C. Bayer, *Alyogyne huegelii* (Endl.) Fryxell, *Pavonia schiedeana* Steud.) shifted into clade III. Additional rearrangements, such as altered relationships among *Adansonia*, *Scleronema* Benth, and *Rhodognaphalon* (Ulbr.) Roberty (clade I) and the placement of *Pavonia*, *Decaschistia*, and two *Hibiscus* species in clade II (**Supplementary Figure 4**; **Supplementary Table 4**).

Comparison with the rDNA tree revealed similar patterns: multiple species from clades I and II were grouped into clade III, with further positional changes within clades (e.g., *Bombax ceiba*, *Fremontodendron*, and *Rhodognaphalon* in clade I; *Howittia*, *Hibiscus*, and *Decaschistia byrnesii* Fryxell in clade II) (**Supplementary Figure 4**; **Supplementary Table 4**).

### Diversity of repeats and correlation with chromosome number

**Figure 4:**
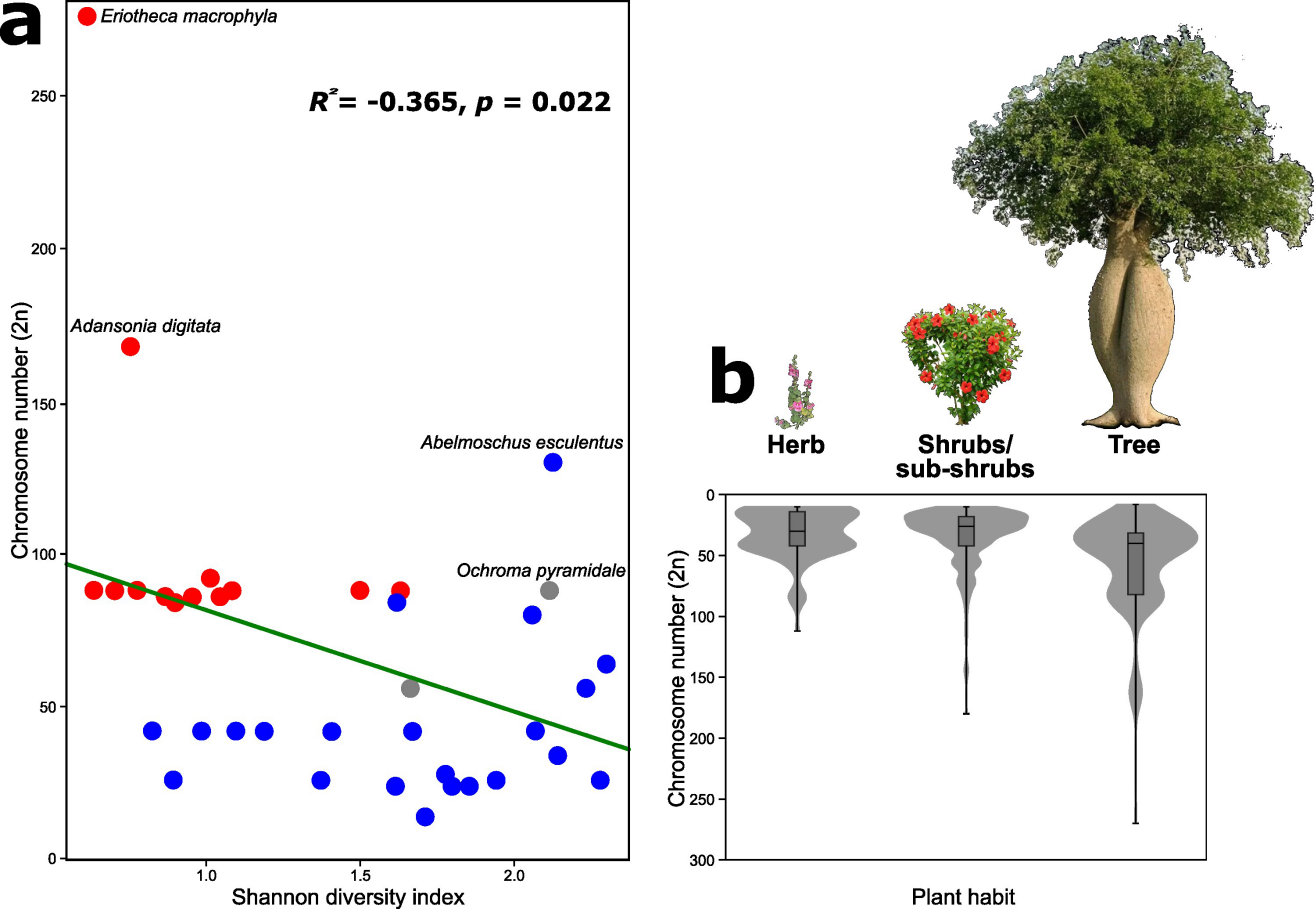
Repeat diversity and chromosome number variation in Malvaceae. **(a)** Shannon diversity index of repetitive DNA elements in Malvatheca species, colored by taxonomic group: Bombacoideae (red), Malvoideae (blue), and incertae sedis (gray). **(b)** Chromosome number distributions across growth habits (herbs, shrubs/subshrubs, and trees) compiled from Malvaceae species.

The repeat diversity of Malvatheca, as measured by the Shannon (H) and Simpson (D) indices, ranged from very low values in *Rhodognaphalon schumannianum* (A. Robyns) A. Robyns (H = 0.4, D = 0.17) to high values in *Hibiscus brachysiphonius* F. Muell. (H = 2.4, D = 0.9). Malvoideae species generally presented greater repeat diversity (H = 0.8–2.4; D = 0.3–0.9) than did Bombacoideae, which tended toward lower values (**Figure 4a**; **Supplementary Table 5**). Chromosome number was negatively correlated with repeat diversity (Shannon: R = –0.36, p = 0.022; Simpson: R = –0.39, p = 0.011), indicating that species with higher chromosome counts, such as Bombacoideae, typically presented fewer diverse repeatomes, whereas Malvoideae (lower 2n) presented greater diversity (**Figure 4b**; **Supplementary Table 5**).

To assess whether chromosome number is correlated with growth habits, we compiled data from 84 Malvaceae species. Trees presented consistently greater chromosome numbers than shrubs and herbs did (range: 8–328; median = 30), supporting an association between the arboreal habit and large chromosome complements (**Figure 4b**; **Supplementary Table 6**).

## Discussion

### Would the diversification of Malvatheca be driven by reticulate evolution?

Our data provides phylogenetic evidence for RE in the Malvatheca clade, corroborating previous genomic analyses (Karimi et al., 2020; Wan et al., 2024; Zhang et al., 2025). These reticulation events appear to have occurred on different timescales (Hernández-Gutiérrez et al., 2022; Yang et al., 2025; Stull et al., 2023), ranging from more recent lineages (e.g., the 21.6 Mya genus *Adansonia*; Wan et al., 2024) to the 53.5 Mya origin of Bombacoideae (Zizka et al., 2020). Hybridization has long been recognized as a fundamental evolutionary force shaping plant diversity, with impacts ranging from immediate reproductive isolation to long-term adaptive radiation (Rieseberg, 1997; Soltis et al., 2004; Chester et al., 2012). Phylogenetic analysis of Malvaceae revealed a complex reticulated evolutionary history. Network inference (PhyloNet) under Maximum Parsimony and Maximum Likelihood criteria rejected strict bifurcation in favor of a network with three hybridization events. The clades III and IV were consistently associated with reticulation points in both plastid and nuclear datasets, suggesting a complex hybrid origin (Hernández-Gutiérrez et al., 2022; Yang et al., 2025). The morphological and cytogenetic heterogeneity of its members supports this view. Sampling the only three genera of Matisioideae (*Matisia*, *Quararibea*, and *Phragmotheca*) allowed us to demonstrate that most representatives of the subfamily (except *Q. funebris*) are grouped into a well-supported clade. The hybrid origin test of this clade, involving a reticulation event between Bombacoideae and Malvoideae, was inconclusive, and our analyses suggest that it may simply be a case of high levels of deep incomplete lineage sorting (ILS) among these subfamilies. Regarding the methods used here to calculate hybridization or introgression (D-statistics), it is worth noting that these tests can be highly sensitive to evolutionary rate variation across different lineages, which can frequently lead to false positives or inconclusive results (Frankel and Ané, 2023). Given the phylogenetic, morphological, and systematic complexity of the relationship between the Malvaceae subfamilies (Colli-Silva et al., 2025), these hypotheses of reticulated evolution need to be tested further with a larger sample size. There are no widely sampled phylogenetic hypotheses for the genus *Quararibea*; therefore, the unexpected position of *Q. funebris* would need to be investigated in more detail, and may be related to taxonomic identification error, reticulation, and/or methodological limitations (e.g., low coverage).

While nuclear low copy genes, rDNA and plastid datasets provided strong phylogenetic resolution for Malvatheca, the repeat-based approach (Dodsworth et al., 2015) failed to resolve relationships. This loss of signal may reflect (i) the impact of reticulation, as hybridization, particularly in allopolyploid contexts, can induce substantial restructuring of repetitive elements through sequence loss, differential retention of parental repeats, and epigenetic reorganization, thereby altering repeat presence, absence, and relative abundance (Parisod et al., 2010), and/or (ii) the deep divergence of Bombacoideae (53.5 Mya; Zizka et al., 2020). Because repeats undergo rapid evolutionary turnover, their phylogenetic utility can erode in ancient groups. Nevertheless, recent studies have shown that repeatome composition can retain signals and even capture ancient hybridization through characteristic proliferation patterns (Castro et al., 2024; Hlavatá et al., 2024). The contrast across plant groups highlights the lineage- and timescale-specific nature of repeat data. For example, in *Erythrostemon* Klotzsch (Leguminosae, 33.6 Mya), systematic variation in repeat composition revealed discordant profiles consistent with ancient origins (Castro et al., 2024). Similarly, in *Amomum* L. (Zingiberaceae, 19.3 Mya), bursts of repeat proliferation coincided with inferred hybridization events, driving genome size changes that track clade divergence (Li et al., 2023; Hlavatá et al., 2024).

Genomic-scale phylogenies further demonstrated that reticulate evolution is pervasive across Malvaceae, challenging tree-based models (Hernández-Gutiérrez et al., 2022; Yang et al., 2025; Zhang et al., 2025). Using 268 nuclear loci across 96 genera, Hernández-Gutiárrez and Magallon (2019) detected extensive discordance attributable to incomplete lineage sorting and introgression, showing that bifurcating trees fail to capture the family’s evolutionary complexity. Reticulation manifests at multiple scales, from recent interspecific gene flow to ancient allopolyploid events. Hyb-Seq datasets and network inference have revealed well-supported introgression in baobabs (*Adansonia*), clarifying floral homoplasy and pollination biology (Karimi et al., 2020). Similarly, independent hybridization and introgression across *Gossypium* highlight the role of allopolyploidy in diversification (Zhang et al., 2025). Cytonuclear processes in *Hibiscus* (Pfeil, 2002) involve chloroplast capture, whereas cytogenetic work in *Eriotheca* (Serra et al., 2022) confirms that hybridization and polyploidy remain active processes. Together, these findings underscore that phylogenetic networks, rather than bifurcated trees, more accurately represent Malvaceae evolution and highlight the central role of RE in shaping its genomic and phenotypic diversity.

### The repeatome compositions of the Malvatheca clade subfamilies

Genomic studies indicate that the Malvatheca clade has undergone multiple rounds of polyploidization, including reticulate allopolyploidy and subsequent dysploidy, which have played central roles in its diversification (Hernández-Gutiérrez et al., 2022; Yang et al., 2025; Zhang et al., 2025). Recent chromosome-scale assemblies of *Adansonia* spp., *Bombax ceiba* and *Ceiba pentandra* have provided unprecedented insights into genome structure and repeat composition across Malvaceae (Wan et al., 2024; Shao et al., 2024). Complementary phylogenomic studies, such as those on *Hibiscus* L., have revealed at least three independent whole-genome duplication (WGD) events (Eriksson et al., 2021), highlighting the recurrent nature of polyploidy within the family. These duplications supplied raw genetic material for evolutionary novelty, whereas subsequent post-polyploid diploidization processes reshaped karyotypes and contributed to current genomic diversity patterns.

The broader evolutionary significance of WGD has been well documented across angiosperms. Large-scale comparative studies have shown that WGDs are not randomly distributed in time but cluster during episodes of environmental stress (Van de Peer et al., 2017; Teng et al., 2022). This has given rise to the so-called “polyploidy paradox”: although WGD frequently occurs, only a subset of polyploid lineages survive in the long term (Van de Peer et al., 2021). Several of these surviving events appear to coincide with the Cretaceous–Paleogene (K–Pg) boundary (~66 Mya), a period of mass extinction. WGDs at this time are hypothesized to have facilitated lineage survival and diversification by buffering against genomic stress and providing redundancy for adaptive innovation (Fawcett et al., 2009; Baduel et al., 2018; Bomblies, 2020). The “polyploid hop” model further proposes that polyploidy creates both challenges and opportunities: initial barriers to establishment may be overcome by long-term adaptive potential (Baduel et al., 2018). Recent studies also suggest that specific life-history traits, such as seed biology, may interact with polyploid status to influence which lineages persist across the K–Pg boundary (Cai et al., 2019; Berry and Jaganathan, 2022).

Within Malvaceae, repeatome dynamics provide important clues to post-WGD genome evolution. Chromosome-scale assemblies have shown that transposable element (TE) proliferation and chromosome restructuring are major forces shaping genome size and karyotype variation (Li et al., 2024; Shao et al., 2024). In Malvatheca, distinct repeatome profiles are observed among subfamilies: Bombacoideae tends to exhibit lower repeat diversity, Malvoideae has higher abundance and diversity, and Matisioideae has intermediate patterns. These differences likely reflect contrasting evolutionary strategies for restructuring genomes after polyploidy, with Bombacoideae favoring karyotypic stability through TE amplification, Malvoideae accumulating a more diverse repeat fraction, and Matisioideae retaining a mixed profile.

Taken together, these patterns suggest that the interplay among ancient WGDs, repeatome evolution, and post-polyploid diploidization has been a major driver of lineage-specific diversification in Malvatheca. Variation in repeat composition and chromosome number across subfamilies reflects different genomic strategies for managing the legacy of polyploidy, highlighting the central role of repetitive DNA in shaping both the stability and flexibility of plant genomes over deep evolutionary timescales.

### The habit correlated with chromosome number

Our results revealed that tree-dominated lineages presented the highest chromosome numbers. In Bombacoideae, striking examples include *Pseudobombax* and *Pachira* (2n = 88–92), *Adansonia digitata* (2n = 160), and *Eriotheca* species, with exceptionally elevated counts of 2n = 194–276 (Costa et al., 2017). A similar pattern was described for incertae sedis, such as *Ochroma pyramidale* (2n = 84) (Costa et al., 2017). A similar trend occurs in the exclusively arboreal Tilioideae, where counts range from 2n = 82 to 2n = 328 (Pigott, 2002; 2012). These data reflect the trend toward ancient whole-genome duplication (WGD) events, followed by the lineage-specific conservation of high chromosome numbers in long-life-cycle species.

Shrubby lineages, such as *Hibiscus* (2n ≈ 52) and *Pavonia* (2n ≈ 56), present intermediate values, possibly representing transitional stages between herbaceous and woody forms. In contrast, herbaceous genera such as *Sida* are chromosomally conserved, typically ranging from 2n = 14–32 (Skovsted, 1935; Fernández et al., 2003). These reductions likely result from dysploidy — the step-wise loss or fusion of chromosomes — a process often associated with short-lived, fast-reproducing life cycles (Siljak-Yakovlev et al., 2017).

The habit–chromosome relationship thus reflects contrasting evolutionary pressures. In woody clades such as Bombacoideae, ancient polyploidization established high baselines that were further expanded by more recent polyploidy (Marinho et al., 2014). Herbaceous lineages, in turn, underwent systematic reductions, which is consistent with patterns observed in other families, such as Asteraceae, where perennial-to-annual transitions are linked to smaller karyotypes (Watanabe et al., 1999). Selective pressures on herbaceous species — favoring rapid cell cycles and short generation times — likely reinforced this reduction. Similar correlations between life form and karyotype have been documented across angiosperms (e.g., Malpighiaceae, Lombello and Forni-Martins, 2003; Ehrendorfer, 1989), but Malvaceae provides one of the clearest examples: Bombacoideae, dominated by trees, consistently carries higher chromosome numbers than Malvoideae, which include mostly herbs and shrubs. These results suggest that major life form transitions are accompanied by fundamental changes in genome organization.

## Conclusions

Our phylogenomic analyses provide strong evidence for reticulate evolution in the Malvatheca clade (Malvaceae), underscoring its role as a fundamental driver of diversification in the family. Ancient hybridization events, coupled with allopolyploidy and subsequent diploidization, have left lasting imprints on the genomic architecture and subfamily relationships. Nuclear and plastid datasets revealed extensive reticulation, whereas repeatome analyses proved less effective for deep phylogenetic resolution, reflecting rapid lineage-specific turnover. This contrast highlights the importance of multilayered genomic approaches for reconstructing complex evolutionary histories. Comparative genomic and cytogenetic evidence also reveals subfamily specific patterns in karyotype evolution. Tree-dominated lineages, particularly Bombacoideae, exhibit exceptionally high and variable chromosome numbers arising from successive polyploidization, whereas herbaceous taxa display reduced counts through dysploidy and genome downsizing. These correlations suggest a close interplay between life form, polyploidy, and genome structure: woody lineages preserve high baselines established by ancient WGDs, whereas herbaceous taxa evolve toward reduced karyotypes under different life-history constraints. Taken together, our results demonstrate how hybridization, polyploidy, and repeat dynamics have jointly shaped Malvaceae diversification. This family offers a compelling model for understanding the integration of reticulate processes, structural genome evolution, and life-history strategies in driving morphological and ecological innovation. Future research incorporating chromosome-scale assemblies and functional studies will be critical for disentangling the genomic consequences of WGD and reticulation across this diverse plant lineage.

## Data Availability

The datasets presented in this study can be found in online repositories. The names of the repository/repositories and accession number(s) can be found in the article/**Supplementary Material**.

## Supplementary material

Supplementary Figure 1 Circular tanglegram comparing phylogenetic topologies inferred from low-copy nuclear genes (Genes), plastid genomes (Plastid), and ribosomal DNA (rDNA) for Malvatheca (Malvaceae). Branches and connecting links are colored according to taxonomic groups: Bombacoideae (red), Malvoideae (blue), Matisioideae (green), and *Incertae sedis* (grey). The central lines connect corresponding taxa between adjacent trees to visualize topological congruence and conflict. Roman numerals (I–IV) indicate major clades recovered in the analyses.

Supplementary Figure 2 Phylogenetic relationships inferred from whole rDNA, plastome, low copy nuclear genes and repeat abundance data. Maximum Likelihood trees for the rDNA, plastome and low copy nuclear genes are shown followed by a Neighbor-Joining tree based on repeat abundance. Support values are shown on nodes for all trees.

Supplementary Figure 3 Phylogeny and repeatome analysis of the Malvatheca clade (Malvaceae). The plastome-based phylogeny (left) shows the evolutionary relationships between species. The heatmap (right) compares the abundance of different repetitive element classes across these species, with brown indicating high abundance and light colors indicating low abundance.

Supplementary Figure 4 Phylogenetic relationships within the Malvatheca clade inferred from inferred from plastome, nuclear rDNA and low copy nuclear genes (left) and repeats abundance (right) sequence data. Tip labels are color-coded by taxonomic group: Malvoideae (blue), Bombacoideae (red), Matisioideae (green), and *incertae sedis* (gray).
