# Supplementary figures and images for: How much can reticulate evolution entangle plant systematics? Revisiting subfamilial classification of the Malvatheca clade (Malvaceae) on the basis of phylogenomics
Source: Front Plant Sci. 2026 Jan 23;16:1717745. doi: 10.3389/fpls.2025.1717745 (PMC12876233; doi:10.3389/fpls.2025.1717745)

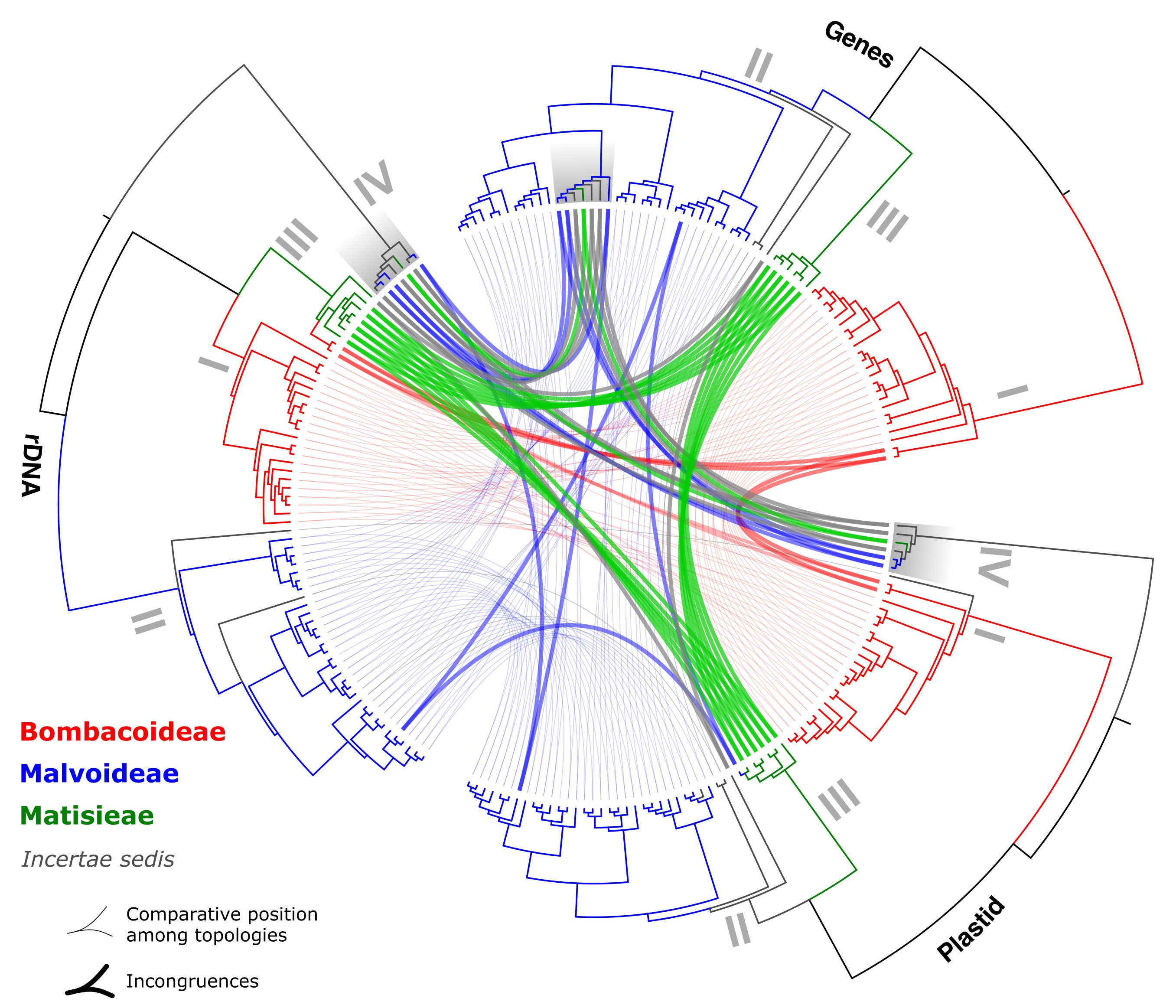

Supplement: Supplementary Figure 1 — Circular tanglegram comparing phylogenetic topologies inferred from low-copy nuclear genes (Genes), plastid genomes (Plastid), and ribosomal DNA (rDNA) for Malvatheca (Malvaceae). Branches and connecting links are colored according to taxonomic groups: Bombacoideae (red), Malvoideae (blue), Matisioideae (green), and Incertae sedis (grey). The central lines connect corresponding taxa between adjacent trees to visualize topological congruence and conflict. Roman numerals (I–IV) indicate major clades recovered in the analyses. [file Image1.jpeg]

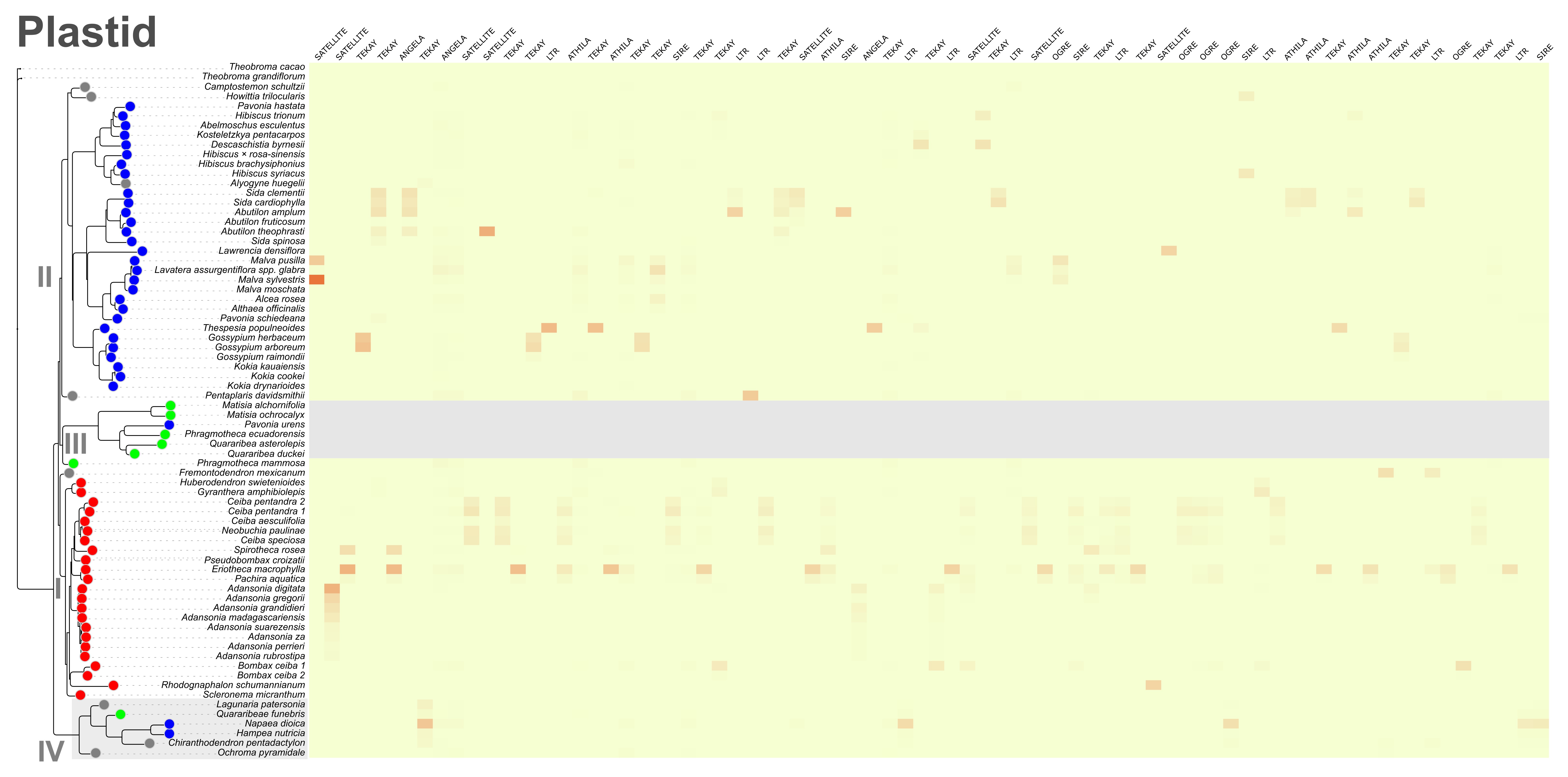

Supplement: Supplementary Figure 3 — Phylogeny and repeatome analysis of the Malvatheca clade (Malvaceae). The plastome-based phylogeny (left) shows the evolutionary relationships between species. The heatmap (right) compares the abundance of different repetitive element classes across these species, with brown indicating high abundance and light colors indicating low abundance. [file Image3.jpeg]

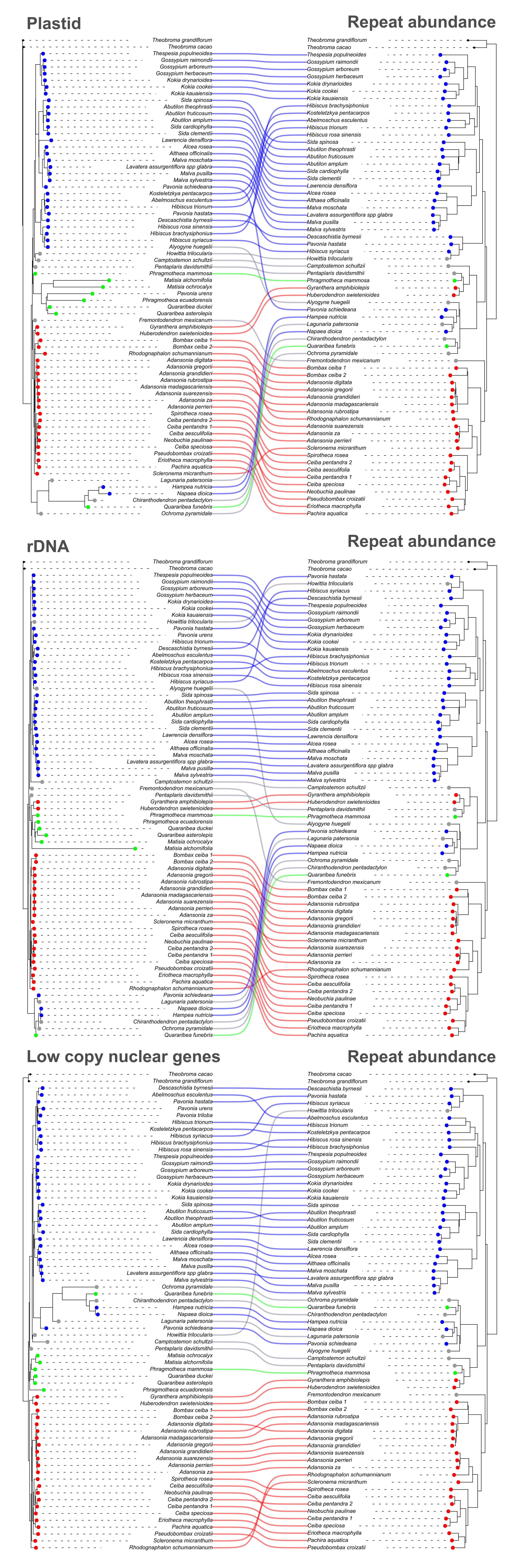

Supplement: Supplementary Figure 4 — Phylogenetic relationships within the Malvatheca clade inferred from inferred from plastome, nuclear rDNA and low copy nuclear genes (left) and repeats abundance (right) sequence data. Tip labels are color-coded by taxonomic group: Malvoideae (blue), Bombacoideae (red), Matisioideae (green), and incertae sedis (gray). [file Image4.jpeg]
